# Early motor signs of attention-deficit hyperactivity disorder: a systematic review

**DOI:** 10.1007/s00787-019-01298-5

**Published:** 2019-02-23

**Authors:** A. Athanasiadou, J. K. Buitelaar, P. Brovedani, O. Chorna, F. Fulceri, A. Guzzetta, Maria Luisa Scattoni

**Affiliations:** 1 Research Coordination and Support Service, Istituto Superiore di Sanità, Viale Regina Elena 299, 00161 Rome, Italy; 2 Department of Cognitive Neuroscience, Radboud University Medical Centre, Nijmegen, The Netherlands; 3 Karakter Child and Adolescent Psychiatry, Nijmegen, The Netherlands; 4 IRCCS Stella Maris, Scientific Institute of Child Neurology and Psychiatry, Calambrone, Pisa Italy; 5 Department of Developmental Neuroscience, IRCCS Stella Maris, Calambrone, Pisa Italy

**Keywords:** Attention-deficit hyperactivity disorder (ADHD), Early motor signs, Infancy, General movements (GMs)

## Abstract

ADHD is a common neurodevelopmental disorder with onset of symptoms typically in early childhood. First signs of the disorder, including language delay, motor delay and temperament characteristics, may be evident as early as infancy. The present review describes published evidence about early motor signs of either children with later symptoms of ADHD or a later diagnosis of the disorder. Nine published cohort studies were included after a systematic search of related terms in PubMed and PsycInfo databases. Study eligibility criteria included: (1) report on early motor function or any motor-related signs; (2) the presence of a participants’ assessment by/at 12 months of age; (3) report of a later presence of ADHD symptoms. The limited number of reports included suggests an association between mild early neurological markers and later developmental coordination disorder and motor overflow movements. Unfortunately, due to their small sample sizes and focus on group reports rather than individuals, they have limited power to find strong associations. Early motor indicators of ADHD, if present, appear to be non-specific, and therefore not yet useful in clinical screening. Spontaneous motility seems to be a promising measure for early ADHD detection, although further studies with large cohorts are recommended to determine its clinical role in children at risk for ADHD.

## Introduction

ADHD is a common neurodevelopmental disorder with symptoms typically emerging during early school years and a worldwide prevalence estimated between 5 and 7% [1, 2]. ADHD is characterized by a persistent pattern of inattention and/or hyperactivity–impulsivity which hinders adaptive functioning or compromises development [3]. To be diagnosed with ADHD, symptoms of the disorder must be observed in two or more settings and have negative effects on fundamental aspects of the child’s daily activities. Co-occurring psychiatric conditions are frequently observed, including oppositional defiant disorder (ODD), conduct disorder, anxiety disorders, depression, autism spectrum disorder (ASD) and learning disabilities [4–9]. Children with ADHD also often face difficulties in everyday life, including in their social relationships, academic performance and achievements, and low self-esteem [10]. In addition, they may experience deficits in visuospatial and verbal working memory, vigilance, inhibitory control and planning, problems with coordination of gross and fine motor functions, sequencing of movements [11], difficulties with working memory and self-regulation of emotions, language and speech deficits, arousal and activation and temporal information processing and timing [11–17].

Investigating early motor signs during the first year of life could be of high importance for the study of early biomarkers of common neurodevelopmental disorders, such as ADHD and ASD, which may share neurobiological underpinnings [18–21]. There is evidence that children with ADHD have worse gross motor and fine motor skills than their typically developing peers [22]. Two hypotheses on the source of the motor disadvantages in individuals with ADHD have been put forward. The first hypothesis attributes these motor abnormalities to the core triad of ADHD symptoms: hyperactivity, impulsivity and inattention. According to this theory, inattention [22] and vigilance problems [23] affect motor skill development. The second hypothesis attributes the motor delays to a likely presence of a comorbid disorder such as developmental coordination disorder (DCD) or ASD [23, 24]. Overall, research indicates that attention and impulse control are strongly predictive of gross and fine motor skill development in children with ADHD [24].

The neurobiological basis for the primary theory is that delays in brain maturation are associated with delays in motor development and specific motor skills [25] in the ADHD population. More specifically, motor control and executive function rely on the integrity of the thalamus, known to be affected in individuals with ADHD [26]. In addition, cortical thickness structure abnormalities and hypoactivation in the right globus pallidus, the right frontal cerebellum and frontal region, reported to be present in ADHD, are responsible, among other functions, for precise motor control.

The second theory—of the comorbidity between ADHD and DCD as the potential cause of motor delays—is also supported by neurological findings. Although the comorbidity of ADHD and DCD is not often taken into account, a high percentage of children with ADHD (30–50%) experiences co-occurring DCD with a familial correlation of 0.38 [27–31]. Almost half of individuals with ADHD (34% out of 63%) have been reported to show motor difficulties within the DCD range, particularly in manual dexterity. These difficulties result in low self-esteem and reduced popularity in children [29, 30]. At present, there is evidence that a dopamine-induced imbalance of basal ganglia neuro circuits could also be involved in the underlying neurobiological mechanisms [32, 33]. Thus, health care professionals should be aware of the high prevalence of this co-occurring motor condition.

Cerebellar abnormalities in children with DCD could also explain postural control and balance problems. Children with ADHD without co-occurring DCD have shown fine motor fluency and flexibility, but when a co-occurring DCD condition is present, fine motor difficulties are observed [34]. However, few studies have focused on brain region atypicalities in ADHD children with co-occurring DCD. McLeod et al [35]. found that these children have increased functional connectivity between the primary motor cortex and brain regions involved in motor control, and claimed this is fundamental for their ability to organize and successfully execute movement [36]. However, motor abnormalities in ADHD cannot be attributed only to the co-occurence with DCD, since children with ADHD without DCD do also have motor difficulties, although these are less prominent [37].

Since ADHD symptoms usually emerge during the early school years, both clinical and neurobiological research have focused on school-aged children, adolescents and adults. Interest in early signs of ADHD is, however, rapidly growing. Recent studies report initial evidence of some indicators appearing prior to school-age, including difficult temperament, and language and motor delay [38, 39]. Still, a little is known about whether early signs of ADHD can be reliably observed during the first year of life. This may be partly due to the relative immaturity of cognitive functions related to sustained attention and focused activity during the first months of life, and to the consequent difficulty in reliably assessing them. Increasing evidence suggests that specific motor behaviors observed during the first months of life may be a marker of neurodevelopmental disorders, which show clinical and genetic overlap with ADHD [40]. Some authors suggest that increased activity in infancy could be considered an early sign of ADHD [38, 41–45]. However, other researchers argue that the quality of movements in infancy per se does not predict the disorder [46–48].

To shed light on early motor signs in ADHD and their emergence, we systematically reviewed the publications investigating motor behavior during the first year of life in infants who later develop subclinical ADHD symptoms or are diagnosed with the disorder.

## Methods

A systematic literature search was performed in PubMed and PsycInfo databases including the following keywords: (1) “ADHD” OR “Attention deficit hyperactivity disorder” OR “Attention deficit-hyperactivity disorder”; (2) AND “infant*” OR “infancy” OR “neonatal” OR “newborn” OR “baby”; AND “movement*” OR “motor” OR “sensory-motor” OR “sensori-motor” OR “motion”. A systematic review of the references of the included papers was also performed to ensure a thorough search. The first search was performed in July 2016, and once more in January 2017, which yielded one additional relevant article.

Study eligibility criteria included: (1) report on early motor function or any motor-related signs; (2) the presence of a participants’ assessment by/at 12 months of age; (3) report of a later presence of ADHD symptoms. The first selection was based on the study titles, as identified by one of the authors (AA). Second, abstracts were independently screened for eligibility by two authors (AA and OC). Two authors (AA and OC) independently performed the data extraction and discussed their findings to reach a consensus. Full texts of potentially relevant papers were read to ascertain whether the study met all selection criteria.

The following data were extracted from the included articles: type of study (e.g., longitudinal, cross-sectional, or case control, both retrospective and prospective), source population (e.g., population-based or hospital referrals), participants’ age range, type and timing of early motor signs, type and timing of ADHD diagnosis (based on the DSM-5) [3], or ADHD-specific symptoms (based on interviews/questionnaires), and the study outcome assessment.

Quality ratings were conducted using a modified Methodological Quality Checklist [49] developed for assessing the methodological quality of both randomized and nonrandomized studies. Two of the authors (AA and OC) performed the quality ratings independently, and when necessary, reached a decision by consensus. Thirteen out of the 27 items of the scale were used in the present study, after removing those that applied only to randomized trials and intervention studies. This modified scale yielded a final rating from 0 to 14 points (see Table 1). The same approach was previously used in a systematic review on ADHD [50].

**Table 1:** Quality ratings of studies

	References								
	Johnson et al. [47]	Jaspers et al. [46]	Jeyaseelan et al. [41]	Hadders-Algra and Groothuis [42]	Hadders-Algra et al. [43]	Butcher et al. [44]	Lemcke et al. [48]	Gurevitz et al. [38]	Jacobvitz and Sroufe [45]
Reporting									
1. Is the hypothesis/aim/objective of the study clearly described?	I	I	I	I	Ι	I	Ι	I?	I
2. Are the main outcomes to be measured clearly described in the “Introduction” or “Method” section?	I	I	I	I	Ι	I	0?	I	I
3. Are the characteristics of the patients included in the study clearly described?	I	I	I	I	Ι	I	I	I	I
5. Are the distributions of principal confounders in each group of subjects to be compared clearly described?	2	2	2	2	0?	1?	1	1	2
6. Are the main findings of the study clearly described?	I	I	I	I	I	I	I	I	I
7. Does the study provide estimates of the random variability in the data for the main outcomes?	I	I	I	I	0	I	I (CI)	I	I
10. Have actual probability values been reported (e.g., 0.035 rather than < 0.05) for the main outcomes except where the probability value is less than 0.001?	I	I	I	I	I	I	I (≤ 0.01)	I	I (*p* < 0.09)
11. Were the subjects asked to participate in the study representative of the entire population from which they were recruited?	I	I	I	I	I	I	I	0 Unable to determine	I
12. Were those subjects who were prepared to participate representative of the entire population from which they were recruited?	I	I	I	I	I	I	I	0 Unable to determine	I
13. If any of the results of the study were based on “Data dredging”, was this made clear?	I	I	I	I	I	I	I	I	I
14. Were the statistical tests used to assess the main outcomes appropriate?	I	I	I	I	I	I	I	I	I
15. Were the main outcome measures used accurate (valid and reliable)?	I	0 ADHD outcome was based only on the DSM-IV scale (7 items)	0 Possible follow-up bias	I	I	I	I	I	I
16. Did the study have sufficient power to detect a clinically important effect?	I (80%)	0 No calculation of the sample size	0 Small sample size	0 No calculation of the sample size	0 Small sample size	0 Small sample size	I?	I	0 Small sample size. Not calculated
Quality rating score									

Yes = I, No = 0, Unable to determine = 0

## Results

**Fig. 1:**
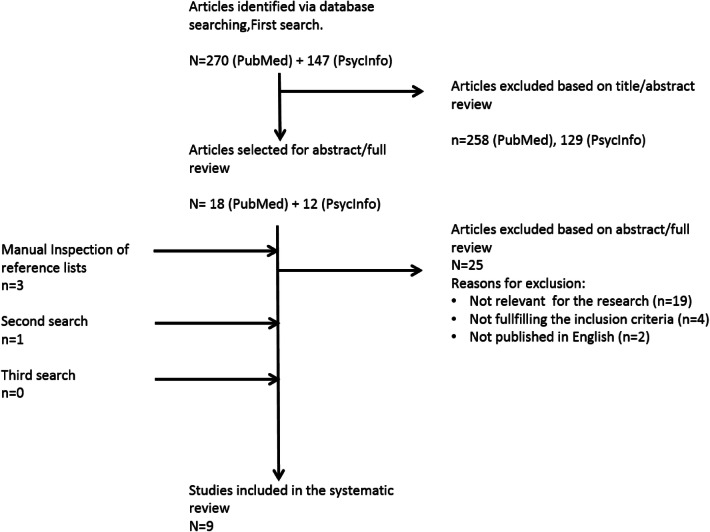
Selection process

In total, 417 articles were identified via the database search on both PubMed and PsycInfo; 30 studies were selected for review. Nine articles were included after completing the selection process (see flow diagram in Fig. 1). All included publications were cohort studies. The findings of all reviewed articles are reported in Table 2. Design and outcome measures differed substantially among the studies, which made a formal meta-analysis not feasible. The quality ratings of the included studies ranged between 11 and 14 out of 14 (see Table 1). Overall, the reports were of good quality.

**Table 2:** Studies included in the current systematic review

Study	Population	Participants	Study design	Early marker (EM) (*n* variables)	Age at EM	Outcome measures (OM)	Age at OM	Summary of results
Johnson et al. [47]	Community-based, within ALSPAC	16 ADHD, 120 controls	Retrospective longitudinal	Body movements (14)	12 months	ADHD diagnosis based on DSM-IV	7 years	No correlation between motion variables at 12 months and ADHD diagnosis at 7 years
Jaspers et al. [46]	Dutch pre-adolescents	348 adolescents with ASD, 419 adolescents with ADHD	Prospective cohort (TRAILS) based on PCH setting	Gross motor skills (16), fine motor skills and adaptation (11), communication and social behavior (10)	Birth, 18 months	CSBQ, CBCL, DSM-IV, oriented attention-hyperactivity problem scale, PCH	10–12 years	Good gross motor skills within the first year significantly correlated with development of ADHD
Jeyaseelan et al. [41]	Neonatal patients	45 extremely low birth weight (< 1000 g) and/or very preterm (< 27 weeks GA) infants	Retrospective longitudinal	NSMDA, neurological status, infant movement patterns, postural development and motor responses to sensory input	12, 24 months	CRSR, ADHD-RS	7–9 years	Motor development of ELBW 24 month infants correlated with specific clinical measures of attention at school-age, independent of biological and social factors
Hadders-Algra and Groothuis [42]	Children from the study of Hadders-Algra et al. [51, 52] (cohort of a larger study)	52 children: 28 healthy term infants, 24 at high-risk for neurodevelopmental disorders	Longitudinal	GM complexity, GM variation, GM fluency	2–4 weeks (low-risk group), 1–6 weeks (high-risk group)	Neurological examination and evaluation of behavior by parental questionnaires GPPQ, DSM-IV based questionnaire adapted for ADHD	4–9 years	Mildly abnormal GMs were associated with the development of MND, ADHD and aggression
Hadders-Algra et al. [43]	Neonatal patients, cohort from a larger study (1988–1993)	41 infants:25 low-risk full-term infants, 16 infants at high-risk for neurodevelopmental disorder	Follow-up prospective	Two forms of normal GMs (normal–optimal, normal–suboptimal) and two forms of abnormal (mildly and definitely abnormal)	Multiple ages during the first postnatal months	ADHD diagnosis, TRF questionnaire based on DSM-IV, CBCL	4–9 years, 9–12 parents completed CBCL and DSMq	Abnormal GMs at ‘writhing’ and ‘fidgety’ age are linked to ADHD along with a psychiatric comorbidity, but no isolated ADHD
Butcher et al. [44]	Neonatal intensive care unit patients (1993–1998)	65 infants born at ≤ 33 gestational weeks	Follow-up	Spontaneous movement (GMs) quality as an indicator of intelligence and behavior at school-age	4–6 week intervals between birth, and at 6 months post-term	Neurological status assessed with Touwen’s test, CBCL with a separate subscale for attention problems	7–11 years (intelligence and behavior)	Spontaneous movements at 11–16 weeks seem to predict both motor development and intelligence. FM quality is strongly associated with later neurological dysfunction. No significant correlation with attention problems
Lemcke et al. [48]	Large population-based birth cohort from a network database	2034 singletons from Danish health registers with a clinical diagnosis of ADHD	Retrospective	Activity and motor development, mothers interviewed about child’s development and temperament	Birth, at 6 and 18 months	ADHD diagnosis based on DSM-IV	8–14 years old	Mothers of children later diagnosed with ADHD reported more or less activity along with a limited cautiousness and a delay in language development. Majority of the children did not show deviations in motor development. There were early and late achievers of independent walking
Gurevitz et al. [38]	Children followed up in well-baby-care clinics	58 children diagnosed with ADHD, 58 typical children	Retrospective	Gross and fine motor (general tone, head and neck control, hyperlaxity of ligaments, physical/neurological abnormalities), language and speech, and cognition and communication	0–1, 3, 9 and 18 months	ADHD diagnosis, family, perinatal and postnatal history, biometric parameters of the infant/toddler, DDST, common difficulties during the first 3 months, sleep and feeding problems, child temperament description by parents and pediatricians, behavior characteristics, abnormal findings on physical examination	Early childhood	Motor (hypotonia and lax ligaments) and language development delay, along with difficult temperament
Jacobvitz and Sroufe [45]	cohort from a larger study of 267 families	34 hyperactive (24 males, 10 females), 34 control children (24 males, 10 females)	Longitudinal	Neonatal status (orientation, arousal, motor maturity, physical ability/body tonus and quieting/consolability), newborn ratings, Carey Infant Temperament Questionnaire, EASI Temperament Survey, home and laboratory observations	Day 7, day 10, 6 months	Teachers completed the Achenbach “Child Behavior Checklst” at the end of kindergarten	5 or 6 years	Hyperactive kindergarteners were less motorically mature on the 7/10 day (isolated finding). Only one out of 38 variables differentiated hyperactive children from controls

*ADHD* attention-deficit hyperactivity disorder, *ASD* autism spectrum disorder, *TRAILS* tracking adolescents’ individual lives survey, *PCH* preventive child healthcare, *CSBQ* children’s social behavior questionnaire, *CBCL* child behavior checklist, *ESSENCE* syndromes eliciting neurodevelopmental clinical examinations, *IDD* intellectual developmental disorder, *DCD* developmental coordination disorder, *NTR* Netherlands twin register, *SES*: socioeconomical status, *ELBW* extremely low birthweight, *NSMDA* neurosensory motor developmental assessment, *GMs* general movements, *MND* minor neurological dysfunction, *DBNC* Danish national birth cohort

### Spontaneous movements during the first 3 months of age

Three prospective studies [42–44] explored very early motor signs of ADHD. They focused on the quality of spontaneous motility, as assessed by the General Movements (GMs) approach with infants at risk for neurodevelopmental delays. General movements are distinct spontaneous movement patterns that infants exhibit without external stimulation [53]. Investigations of early motor indicators of ADHD through the evaluation of GMs have included both healthy infants and those with increased risk for neurodevelopmental delays. Consistent with the GM method, the investigators evaluated infants several times in the first months of life and then followed up with standardized behavioral assessments at school-age. One study [42] reported that infants with definitely abnormal GMs including extremely reduced complexity, variability and fluency were at significantly increased risk to develop cerebral palsy. Furthermore, a significant association was found between milder GM abnormalities and attention problems at 4–9-year follow-up (odds ratio 6.88, 95% CI 1.39–33.97) assessed by the DSM-IV ADHD questionnaire. In particular, unlike infants with normal fidgety movements at 3–4 months, children with mildly abnormal GMs were significantly more distractible, inattentive and hyperactive as assessed by the Groningen Perinatal Project Questionnaire (GPPQ) and the DSM-IV ADHD Questionnaire for Attention-Deficit/Hyperactivity Disorder. Another study [40] indicated that abnormal GMs at both writhing and fidgety age were significantly associated with the presence of ADHD only when it was co-occurrent with another psychiatric diagnosis, but not when it was present in isolation. Furthermore, abnormal GMs at fidgety age were related to a higher total score on the DSM-IV ADHD questionnaire, and in particular, to higher subscores for hyperactivity and impulsivity, and lower subscores for inattention. Another report of GMs with preterm born infants, however, showed no significant association between GMs and attention problems at 7–11 years, as assessed by a separate subscale of the Child Behavior Checklist (CBCL) [55]. This dissociation was even stronger when children with cerebral palsy were excluded from the analysis [44].

### Motor signs during the first year

Of the included reports, four were large longitudinal cohort studies exploring early neurodevelopment in the general population [38, 46, 48] or in families with lower socioeconomic status [45]. Neurodevelopmental characteristics of children with ADHD symptoms or an ADHD diagnosis were compared to the same characteristics of control children.

A retrospective chart review study of 58 children diagnosed with ADHD at school-age and 58 controls that participated in a population-based developmental program at a ‘Well-Baby’ clinic evaluated longitudinal data from birth, 1-, 3-, 9- and 18-month visits [38]. Higher incidence of emergency caesarian sections, smaller head circumference at 3 months and feeding or sleeping difficulties before 6 months were all identified as early signs significantly correlated with ADHD. However, the only motor-related early sign identified was a delay in gross motor development, as assessed by the Denver Developmental Screening Test (DDST) [54]. A delay in gross motor movements was identified at 9 months of age in 34.5% of the ADHD group compared to 13.8% of the controls. The most reported deviation from typical gross motor development was the refusal to maintain supine position, which led to difficulties in head control, and thus, to general motor development difficulties. The delay was reported to be relatively mild, and attributed to physical characteristics including lax ligaments and hypotonia. Importantly, some children in the ADHD group were early achievers and some were late achievers, with both subgroups reported by the authors as showing “extreme” motor behavior.

A prospective study exploring early development in 267 infants from families with lower socioeconomic status [45] also included a smaller retrospective evaluation of 34 hyperactive children and 34 age-matched controls. The presence of hyperactivity was determined at around 6 years of age from subscores of the teacher-administered CBCL [55]. Thirty-eight child behavior variables were obtained during the first 2.5 years of life including neonatal behavioral assessments, mother-administered Carey questionnaire evaluating temperament, activity and attention, and other ratings of activity at 3 and 6 months of age [56]. Children who were hyperactive in kindergarten had been motorically less mature at 7 days old as assessed by the motor maturity Brazelton factor [57]. However, this was the only variable, out of the 38, which differentiated hyperactive children from typical children.

Another large study by Lemcke et al. [48] included 2034 children with a diagnosis of ADHD, who came from a large population-based cohort from the Danish National Birth Cohort (DNBC). As part of the DNBC, 76,286 mothers were interviewed about their child’s development at 6 and 18 months. Children were followed up between 8 and 14 years of age, when they were assessed for the presence of ADHD based on International Classification of Diseases, 10th Revision (ICD-10) criteria. The interview at 6 months of age explored specific aspects of motor development, such as the infant holding their head straight while being picked up, sitting up while on an adult’s lap, rolling over from back to stomach, crawling on the stomach. When comparing the ADHD group with the total study cohort, the only significant finding in the ADHD group was a higher number of infants who could not sit up straight when put on lap at 6 months (*p* ≤ 0.001).

Similarly, Jaspers et al. [46] studied early indicators of ADHD (and ASD) in a population of 1816 subjects who took part in a prospective cohort study among (pre-)adolescents in the general population. Early indicators were obtained by identifying correlations between routine data from the community pediatric services during the first year of life and ADHD-risk as measured by parent-administered CBCL between 11 and 17 years old. Early motor indicators as assessed by the Van Wiechen scheme [58] explored the scores of gross and fine motor skills and social behavior. This study reports that good gross motor skills within the first year were significantly correlated with the development of ADHD problems.

### Motor signs at 1 year

Two studies explored motor signs at 12 months of age. Johnson et al. [46] studied 16 children with ADHD (based on DSM-IV criteria), and 120 control children. Both groups were extracted from a focus study group within a larger community-based cohort, the Avon Longitudinal Study of Parents and Children (ALSPAC). As part of the ALSPAC focus study group, 1240 infants at 1 year took part in a video-recorded parent–infant interaction in a naturalistic environment [59]. Software was used to track 8 body markers [(nose (N), right (RH) and left hand (LH), right (RE) and left elbow (LE), right (RS) and left shoulder (LS) and pelvis (P)]. Thirteen motion summaries were used to determine robust indexes of motor activity including speed, acceleration along with their variability, acceleration, periodicity and agitation. Finally, 14 out of 104 variables were chosen for further investigation, including the speed and variability of 5 markers (N, RH, LH, LE, LS), the agitation of 3 (N, LH, LE) and rhythmic movement of one marker (RH). No significant association was found between the motion variables examined and the diagnosis of ADHD at 7 years. A correlation between motor activity and scores on the inattentiveness subscale of the ADHD diagnostic interview was found in male participants, but considered questionable by the authors due to the small size of the subsample (*n* = 14).

Lastly, motor signs of extremely low birth weight and very preterm infants were evaluated at 12 months with the Neurosensory Motor Developmental Assessment (NSMDA) and these scores were examined together with clinical and psychometric measures of attention at 7–9 years of age [41]. At 12 months, NSMDA evaluated gross and fine motor function, motor patterns, neurological status, postural development, and the reaction to sensory input. Measures of attention in childhood included the Conner’s Rating Scale Revised-Long Form (CRSR) and Du Paul ADHD Rating Scale IV (ADHD-RS).

## Discussion

ADHD is a neurodevelopmental disorder characterized by a pattern of inattention and/or impulsivity and hyperactivity across different contexts. Since early identification of ADHD is essential to optimize the quality of life, there is growing research interest in the investigation of early clinical and behavioral features of children later diagnosed with ADHD. To further investigate this topic, we reviewed the literature summarizing the full spectrum of motor impairments which might be potential early indicators of ADHD. In particular, we included studies which report motor skills of infants during the first year of life who subsequently (1) received a formal psychiatric diagnosis of ADHD based on DSM-IV or the ICD-10, or (2) whose behaviors were related to high levels of ADHD symptoms, as identified by questionnaires.

### Diagnosis of ADHD and early motor signs

Four of nine studies presented included a formal diagnosis of ADHD through a psychiatric assessment [36, 42, 47, 48]. The clinical diagnosis of ADHD was either based on the criteria of the DSM-IV or the ICD-10. In these studies, children with ADHD showed atypical motor development detectable in the first 9 months [47], but not as late as 12 months, when compared to typically developing infants.

The first detectable abnormalities of motor development, GMs, in children later diagnosed with ADHD seem to be associated more strongly with ADHD when it is co-occurring with other psychiatric disorders than with ADHD alone. This is consistent with previous reports suggesting that ADHD with a co-occurring disorder is a probably more severe form of ADHD [60, 61]. Indeed, although children diagnosed with cerebral palsy were excluded from Hadders-Algra’s [42] study, to avoid bias related to the known association between cerebral palsy and behavioral problems, their study population was at high risk for neurodevelopmental problems [43]. Therefore, the relationship found in this study between abnormal GMs and ADHD still suggests that the vulnerability of periventricular white matter, typical of preterm subjects and associated to abnormal GMs, may contribute to the development of ADHD with co-occurring conditions [62, 63]. In any case, the results of Hadders-Algra [42] should be considered as preliminary, since its sample size was insufficient to reach definite conclusions [43].

During the time of spontaneous motility (0–5 months) and beyond, at least up to 9 months, a delay in gross motor function was significantly more common in infants who later developed ADHD. At 3 and 9 months, Gurevitz et al. [38] reported a delay in gross motor development as assessed by the Denver Developmental Screening Test, while at 6 months Lemcke et al. [48] found a significantly higher number of infants who could not sit up straight when put on lap in the ADHD group. Motor delay seems to be no longer present at 12 months, according to the findings by Johnson et al. [46], who found no significant association between a series of motor variables at 12 months with the clinical diagnosis of ADHD at 7 years of age. As the authors hypothesized, their inconclusive outcome could be due to the small sample size of the study.

Auerbach et al. [64], examining 7-month-old infants at risk of ADHD based on mother reports and observational measures, found that children with later ADHD were significantly different from the control group in respect of behavioral states, interest and activity level.

Overall, these results support the hypothesis of a link between mild neurological markers and developmental coordination disorder, and motor overflow movements, all of which are more common in children with ADHD [64]. Nevertheless, non-specific factors related to physical characteristics, such as lax ligaments and hypotonia, are also likely to have contributed to the described gross motor delay.

### Symptoms of ADHD and early motor signs

Results are more inconsistent when it comes to the relationship between early motor signs and later subclinical ADHD symptoms. Some reports state that early spontaneous motility is correlated with attention problems, including less motoric maturity at 7 days of life in children who are later hyperactive in kindergarten. Similarly, Jeyaseelan et al. [41] found a correlation between decreased motor and sensory assessment scale scores (NSMDA) and psychometric measures of verbal attention span at 12 months. However, Jaspers et al. [46] found that ADHD problems were significantly correlated with good gross motor skills, as defined by the authors, within the first year of life.

There are multiple possible reasons for these inconsistent findings. The populations that were studied are extremely heterogeneous and have different degrees of risk for ADHD, from infants with clinical signs of early hyperactivity to those who are born preterm or were small for gestational age. The assessments used to test the presence and characteristics of early motor signs were also heterogeneous, including parental questionnaires, qualitative and quantitative assessments of motor behavior, and early attentional measures. Finally, the diagnostic instruments that were used to evaluate later presence of ADHD symptoms differed among studies, making comparisons very challenging.

The limitation of our review is that the studies which are included have small sample sizes and focus on group reports rather than individuals, so they have limited power to find strong associations. Although the studies are of high quality according to the rating scheme, the outcome measures have questionable accuracy (see Table 1).

### Concluding remarks

In summary, there are a limited number of reports investigating gross motor function in the first year of life in children who later have ADHD symptoms or are diagnosed with ADHD. Early detection of ADHD-related motor abnormalities would be important to provide a timely diagnosis, and most importantly, early intervention, especially in case of a very strong association between ADHD and early motor signs. This would assist clinicians in the continuous development and implementation of interventions at a very critical period of child development, when the brain is rapidly developing and neuroplasticity is highest. Unfortunately, data emerging from this review show that early motor signs, if present, seem to be non-specific, and therefore not yet worth implementing in clinical screening protocols. Some qualities of spontaneous motility seem promising as an early detection tool for risk of ADHD, although further studies based on the individual, with larger cohorts and more specific and semi-quantitative scoring systems, are necessary to determine their clinical role in populations at risk for ADHD.

## References

[1] Polanczyk G, Rohde LA (2007). Epidemiology of attention-deficit/hyperactivity disorder across the lifespan. Curr Opin Psychiatry.

[2] Willcutt EG (2012). The prevalence of DSM-IV attention-deficit/hyperactivity disorder: a meta-analytic review. Neurother J Am Soc Exp NeuroTher.

[3] American Psychiatric Association (2013). Diagnostic and statistical manual of mental disorders, 5th edition (DSM-5).

[4] Alzaben FN, Sehlo MG, Alghamdi WA, Tayeb HO, Khalifa DA, Mira AT (2018). Prevalence of attention deficit hyperactivity disorder and comorbid psychiatric and behavioral problems among primary school students in western Saudi Arabia. Saudi Med J.

[5] Gordon-Lipkin E, Marvin AR, Law JK, Lipkin PH (2018). Anxiety and mood disorder in children with autism spectrum disorder and ADHD. Pediatrics.

[6] Quintero J, Morales I, Vera R, Zuluaga P, Fernández A (2017). The impact of adult ADHD in the quality of life profile. J Atten Disord.

[7] Reimherr FW, Marchant BK, Gift TE, Steans TA (2017). ADHD and anxiety: clinical significance and treatment implications. Curr Psychiatry Rep.

[8] Rimal H, Pokharel A (2016). Prevalence of attention deficit hyperactivity disorder among school children and associated co-morbidities—a hospital based descriptive study. Kathmandu Univ Med J (KUMJ).

[9] Willcutt EG, Pennington BF, Smith SD, Cardon LR, Gayán J, Knopik VS (2002). Quantitative trait locus for reading disability on chromosome 6p is pleiotropic for attention-deficit/hyperactivity disorder. Am J Med Genet.

[10] Faraone SV, Biederman J, Spencer T, Mick E, Murray K, Petty C (2006). Diagnosing adult attention deficit hyperactivity disorder: are late onset and subthreshold diagnoses valid?. Am J Psychiatry.

[11] Barkley RA (1997). Attention-deficit/hyperactivity disorder, self-regulation, and time: toward a more comprehensive theory. J Dev Behav Pediatr JDBP.

[12] Wehmeier PM, Schacht A, Dittmann RW, Banaschewski T (2010). Minor differences in ADHD-related difficulties between boys and girls treated with atomoxetine for attention-deficit/hyperactivity disorder. Atten Deficit Hyperact Disord.

[13] Berlin L, Bohlin G, Nyberg L, Janols L-O (2004). How well do measures of inhibition and other executive functions discriminate between children with ADHD and controls?. Child Neuropsychol J Norm Abnorm Dev Child Adolesc.

[14] Schroeder VM, Kelley ML (2009). Associations between family environment, parenting practices, and executive functioning of children with and without ADHD. J Child Fam Stud.

[15] Ponitz CC, McClelland MM, Matthews JS, Morrison FJ (2009). A structured observation of behavioral self-regulation and its contribution to kindergarten outcomes. Dev Psychol.

[16] Faraone SV, Asherson P, Banaschewski T, Biederman J, Buitelaar JK, Ramos-Quiroga JA (2015). Attention-deficit/hyperactivity disorder. Nat Rev Dis Primers.

[17] Tillman C, Brocki KC, Sørensen L, Lundervold AJ (2015). A longitudinal examination of the developmental executive function hierarchy in children with externalizing behavior problems. J Atten Disord.

[18] Rommelse NNJ, Franke B, Geurts HM, Hartman CA, Buitelaar JK (2010). Shared heritability of attention-deficit/hyperactivity disorder and autism spectrum disorder. Eur Child Adolesc Psychiatry.

[19] Rommelse NNJ, Peters CTR, Oosterling IJ, Visser JC, Bons D, van Steijn DJ (2011). A pilot study of abnormal growth in autism spectrum disorders and other childhood psychiatric disorders. J Autism Dev Disord.

[20] Rommelse N, Antshel K, Smeets S, Greven C, Hoogeveen L, Faraone SV, Hartman CA (2017). High intelligence and the risk of ADHD and other psychopathology. Br J Psychiatry J Ment Sci.

[21] Visser JC, Rommelse NNJ, Greven CU, Buitelaar JK (2016). Autism spectrum disorder and attention-deficit/hyperactivity disorder in early childhood: a review of unique and shared characteristics and developmental antecedents. Neurosci Biobehav Rev.

[22] Ghanizadeh A (2010). Comorbidity of enuresis in children with attention-deficit/hyperactivity disorder. J Atten Disord.

[23] Kaiser M-L, Schoemaker MM, Albaret J-M, Geuze RH (2015). What is the evidence of impaired motor skills and motor control among children with attention deficit hyperactivity disorder (ADHD)? Systematic review of the literature. Res Dev Disabil.

[24] Tseng MH, Henderson A, Chow SMK, Yao G (2004). Relationship between motor proficiency, attention, impulse, and activity in children with ADHD. Dev Med Child Neurol.

[25] Rosa Neto F, Goulardins JB, Rigoli D, Piek JP, de Oliveira JA (2015). Motor development of children with attention deficit hyperactivity disorder. Revista Brasileira De Psiquiatria (Sao Paulo, Brazil: 1999).

[26] Valera EM, Faraone SV, Murray KE, Seidman LJ (2007). Meta-analysis of structural imaging findings in attention-deficit/hyperactivity disorder. Biol Psychiat.

[27] Gillberg C, Gillberg IC, Rasmussen P, Kadesjö B, Söderström H, Råstam M (2004). Co-existing disorders in ADHD—implications for diagnosis and intervention. Eur Child Adolesc Psychiatry.

[28] Fliers E, Rommelse N, Vermeulen SHHM, Altink M, Buschgens CJM, Faraone SV (2008). Motor coordination problems in children and adolescents with ADHD rated by parents and teachers: effects of age and gender. J Neural Transm (Vienna, Austria: 1996).

[29] Fliers E, Vermeulen S, Rijsdijk F, Altink M, Buschgens C, Rommelse N (2009). ADHD and poor motor performance from a family genetic perspective. J Am Acad Child Adolesc Psychiatry.

[30] Fliers EA, de Hoog MLA, Franke B, Faraone SV, Rommelse NNJ, Buitelaar JK, Nijhuis-van der Sanden MWG (2010). Actual motor performance and self-perceived motor competence in children with attention-deficit hyperactivity disorder compared with healthy siblings and peers. J Dev Behav Pediatr JDBP.

[31] Magalhães LC, Missiuna C, Wong S (2006). Terminology used in research reports of developmental coordination disorder. Dev Med Child Neurol.

[32] Arnsten AFT (2006). Fundamentals of attention-deficit/hyperactivity disorder: circuits and pathways. J Clin Psychiatry.

[33] Fliers EA, Vasquez AA, Poelmans G, Rommelse N, Altink M, Buschgens C (2012). Genome-wide association study of motor coordination problems in ADHD identifies genes for brain and muscle function. World J Biol Psychiatry Off J World Feder Soc Biol Psychiatry.

[34] Fong SSM, Tsang WWN, Ng GYF (2012). Altered postural control strategies and sensory organization in children with developmental coordination disorder. Hum Mov Sci.

[35] McLeod KR, Langevin LM, Goodyear BG, Dewey D (2014). Functional connectivity of neural motor networks is disrupted in children with developmental coordination disorder and attention-deficit/hyperactivity disorder. NeuroImage Clin.

[36] Zwicker JG, Harris SR, Klassen AF (2013). Quality of life domains affected in children with developmental coordination disorder: a systematic review. Child Care Health Dev.

[37] Langmaid RA, Papadopoulos N, Johnson BP, Phillips J, Rinehart NJ (2016). Movement scaling in children with ADHD-combined type. J Atten Disord.

[38] Gurevitz M, Geva R, Varon M, Leitner Y (2014). Early markers in infants and toddlers for development of ADHD. J Atten Disord.

[39] Arnett AB, Macdonald B, Pennington BF (2013). Cognitive and behavioral indicators of ADHD symptoms prior to school age. J Child Psychol Psychiatry.

[40] Van Damme T, Simons J, Sabbe B, Van West D (2015). Motor abilities of children and adolescents with a psychiatric condition: a systematic literature review. World J Psychiatry.

[41] Jeyaseelan D, O’Callaghan M, Neulinger K, Shum D, Burns Y (2006). The association between early minor motor difficulties in extreme low birth weight infants and school age attentional difficulties. Early Hum Dev.

[42] Hadders-Algra M, Groothuis AM (1999). Quality of general movements in infancy is related to neurological dysfunction, ADHD, and aggressive behavior. Dev Med Child Neurol.

[43] Hadders-Algra M, Bouwstra H, Groen SE (2009). Quality of general movements and psychiatric morbidity at 9 to 12 years. Early Hum Dev.

[44] Butcher PR, van Braeckel K, Bouma A, Einspieler C, Stremmelaar EF, Bos AF (2009). The quality of preterm infants’ spontaneous movements: an early indicator of intelligence and behavior at school age. J Child Psychol Psychiatry.

[45] Jacobvitz D, Sroufe LA (1987). The early caregiver-child relationship and attention-deficit disorder with hyperactivity in kindergarten: a prospective study. Child Dev.

[46] Jaspers M, de Winter AF, Buitelaar JK, Verhulst FC, Reijneveld SA, Hartman CA (2013). Early childhood assessments of community pediatric professionals predict autism spectrum and attention deficit hyperactivity problems. J Abnorm Child Psychol.

[47] Johnson P, Ahamat B, Mcconnachie A, Puckering C, Marwick H, Furnivall D (2014). Motor activity at age one year does not predict ADHD at seven years: infant motor activity and ADHD. Int J Methods Psychiatr Res.

[48] Lemcke S, Parner ET, Bjerrum M, Thomsen PH, Lauritsen MB (2016). Early development in children that are later diagnosed with disorders of attention and activity: a longitudinal study in the Danish National Birth Cohort. Eur Child Adolesc Psychiatry.

[49] Downs SH, Black N (1998). The feasibility of creating a checklist for the assessment of the methodological quality both of randomised and non-randomised studies of health care interventions. J Epidemiol Community Health.

[50] Buitelaar NJL, Posthumus JA, Buitelaar JK (2015). ADHD in childhood and/or adulthood as a risk factor for domestic violence or intimate partner violence: a systematic review. J Atten Disord.

[51] Hadders-Algra M, Van Eykern LA, Klip-Van den Nieuwendijk AW, Prechtl HF (1992). Developmental course of general movements in early infancy. II. EMG correlates. Early Hum Dev.

[52] Hadders-Algra M, Klip-Van den Nieuwendijk A, Martijn A, van Eykern LA (1997). Assessment of general movements: towards a better understanding of a sensitive method to evaluate brain function in young infants. Dev Med Child Neurol.

[53] Prechtl HF (1990). Qualitative changes of spontaneous movements in fetus and preterm infant are a marker of neurological dysfunction. Early Hum Dev.

[54] Frankenburg WK, Dodds JB (1967). The Denver developmental screening test. J Pediatr.

[55] Achenbach TM (1991). Integrative guide for the 1991 CBCL/4-18, YSR, and TRF profiles.

[56] Carey WB (1970). A simplified method for measuring infant temperament. J Pediatr.

[57] Brazelton TB (1973). Assessment of the infant at risk. Clin Obstet Gynecol.

[58] Wiechen JV (1988). Ontwikkelingsonderzoek op het consultatiebureau. *Werkboek bij het herzien van het Van Wiechenschema*.

[59] Thorpe M (2003). Infant formula supplemented with DHA: are there benefits?. J Am Diet Assoc.

[60] Sprich-Buckminster S, Biederman J, Milberger S, Faraone SV, Lehman BK (1993). Are perinatal complications relevant to the manifestation of ADD? Issues of comorbidity and familiality. J Am Acad Child Adolesc Psychiatry.

[61] Batstra L, Neeleman J, Elsinga C, Hadders-Algra M (2006). Psychiatric morbidity is related to a chain of prenatal and perinatal adversities. Early Hum Dev.

[62] Hadders-Algra M (2004). General movements: a window for early identification of children at high risk for developmental disorders. J Pediatr.

[63] Lüchinger AB, Hadders-Algra M, van Kan CM, de Vries JIP (2008). Fetal onset of general movements. Pediatr Res.

[64] Auerbach JG, Atzaba-Poria N, Berger A, Landau R (2004). Emerging developmental pathways to ADHD: possible path markers in early infancy. Neural Plast.

